# PGA: power calculator for case-control genetic association analyses

**DOI:** 10.1186/1471-2156-9-36

**Published:** 2008-05-13

**Authors:** Idan Menashe, Philip S Rosenberg, Bingshu E Chen

**Affiliations:** 1 Biostatistics Branch, Division of Cancer Epidemiology and Genetics, National Cancer Institute, National Institute of Health, Department of Health and Human Services, Rockville, MD, USA; 2 Department of Mathematics and Statistics, Concordia University, Montréal, Québec, Canada

## Abstract

**Background:**

Statistical power calculations inform the design and interpretation of genetic association studies, but few programs are tailored to case-control studies of single nucleotide polymorphisms (SNPs) in unrelated subjects.

**Results:**

We have developed the "Power for Genetic Association analyses" (PGA) package which comprises algorithms and graphical user interfaces for sample size and minimum detectable risk calculations using SNP or haplotype effects under different genetic models and study constrains. The software accounts for linkage disequilibrium and statistical multiple comparisons. The results are presented in graphs or tables and can be printed or exported in standard file formats.

**Conclusion:**

PGA is user friendly software that can facilitate decision making for association studies of candidate genes, fine-mapping studies, and whole-genome scans. Stand-alone executable files and a Matlab toolbox are available for download at:

## Background

Case-control genetic association studies are increasingly being used in studying the genetic basis of human complex traits [1-3]. Statistical power analyses constitute a key step in the design process of these studies. Power calculations elucidates the actual sample size needed to find a true genotype-phenotype correlation under the study constraints [4]. Indeed, most grants applications for genetic association studies require a power analysis section to justify the research proposal. Alternatively, power analysis can be used to explore possible reasons for equivocal or negative results. Thus, it is an indispensable procedure both for *a priori *and *a posteriori *analyses in genetic association studies.

The principals for power calculation can be found in standard statistical textbooks. Moreover, the scientific literature describes the mathematics of power analyses for a variety of specialized experimental designs [4-6]. Yet, there is limited computer-software to assist scientists in this task [7]. Many commonly used computational tools for genetic studies are oriented towards family-based studies [8-11] and only few have been developed to handle power calculations for case-control studies of single nucleotide polymorphisms (SNPs) in unrelated subjects [12-14]. Since the latter approach is increasingly used, we have developed algorithms and graphical user interfaces (GUIs) to calculate the sample size and the minimum detectable relative risk in genetic case-control studies for dominant, co-dominant, and recessive models of SNPs and SNP haplotypes.

## Implementation

The "Power for Genetic Association Analyses" (PGA) package was developed in Matlab and consists a toolbox of command line functions and three unifying graphical user interfaces (GUIs). Users with a Matlab software can run the three GUIs or the command line functions in Matlab environment. Users without a Matlab license can download and install the compiled versions of the three GUIs that run as stand-alone applications under Windows XP or Vista operating systems.

The program assumes that SNPs are biallelic and in Hardy-Weinberg equilibrium. All statistical tests are two-sided. The GUIs called PGA1 and PGA2 can display up to 9 scenarios simultaneously. Hence, they can be used to identify a robust choice of sample size. The graphs produced by each GUI can be printed or exported as TIF files, and tables of numerical results can be exported as HTML or csv files.

## Results

**Figure 1:**
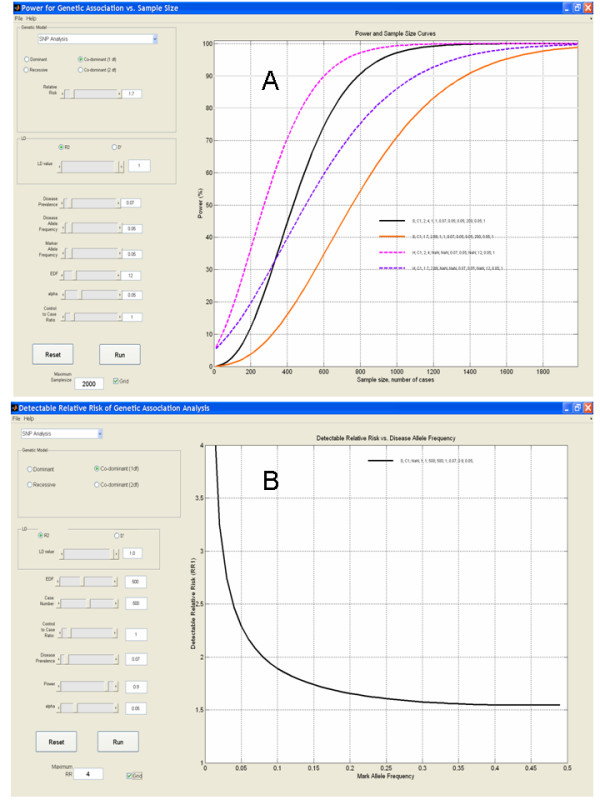
**Graphical user interfaces for statistical power calculations.** (A) PGA1 – statistical power is calculated and plotted for different sample sizes and various genetic and statistical parameters. Input variables (e.g. 'Genetic mode of inheritance', 'disease allele frequency', 'relative risk (RR)', etc.) can be specified using slider controls, or by typing specific values in the corresponding text boxes. Pressing the 'Run' button executes the calculations and plots the relationships between power and sample size according to the specified study parameters. A keyed legend listing the corresponding parameters is shown on the graph. Up to eight different analyses (color-coded) can be displayed simultaneously, allowing the comparison of different scenarios. (B) PGA2 – Minimal detectable relative risk (MDRR) is calculated and plotted for various minor allele frequencies (MAFs) of potential genotyped loci. Input and output is similar to PGA1.

The GUI called PGA1 provides a computational and graphical interface for the relation between statistical power and sample size for dominant, co-dominant and recessive SNP or haplotype effect (Figure 1A). The genotyped markers can include the causative SNP, or be in linkage disequilibrium (LD) with the causal SNP at a given level. The impact of multiple hypothesis testing can be accomplished by adjusting the effective degrees of freedom (EDF) or the alpha level. For example, in a fine-mapping study of 200 effective tests (see below), the sample size required to detect an overall 2-fold increase in risk (assuming a co-dominant model with 1 df) with 90% power, false positive rate of 5%, disease prevalence of 7%, disease allele frequency of 5%, and assuming a complete LD between the genotyped marker and the causative SNP (r^2 ^= 1.0) is 800 cases and 800 controls (Figure 1A). PGA1 allows one to explore the impact of different parameters. For example, reducing the genotype relative risk from 2-fold to 1.7-fold in the same study, increases the required sample size from 800 to 1400 cases and controls. PGA1 is designed to execute power calculations for haplotype data. For example, using the same parameters in the example above and assuming 12 common haplotypes in an LD block within the region show that the required sample size would be 600 and 1100 cases and controls to attain 90% power for relative risks of 2 and 1.7 respectively (Figure 1A).

The GUI PGA2 has a similar interface to PGA1, but it is designed to calculate and plot the minimum detectable relative risk (MDRR) for genetic loci, given a fixed number of cases and controls, according to their minor allele frequencies (MAFs). MDRR can calculate the smallest relative risk that can be detected, with sample in hand, at the target level of power. Hence, PGA2 can assist in designing fine mapping studies of prominent genomic loci, identified from familial linkage analyses or genome-wide association studies. For example, multiple markers along a 600-kb segment on human chromosome 8q24 have recently been associated with prostate cancer susceptibility [15-17]. Consequently, one may want to genotype additional SNPs in this region aiming to find the most strongly associated markers as a prelude to functional or comparative studies. Given a fixed sample size, there is a detection limit such that one is under-powered to detect true associations to SNPs with MAF below a certain threshold. Considerable resources can be saved by excluding SNPs with MAF below the detection threshold. For example, using the PGA2 tool reveals that with a sample size of 500 cases and controls and assuming an effective number of tests (effective degrees of freedom – EDF) of 500, there is no justification (power < 90%) to genotype SNPs with minor allele frequency (MAF) < 0.08 assuming a modest relative risk of ~2-fold as implied by the preliminary studies [15-17] (Figure 1B).

**Figure 2:**
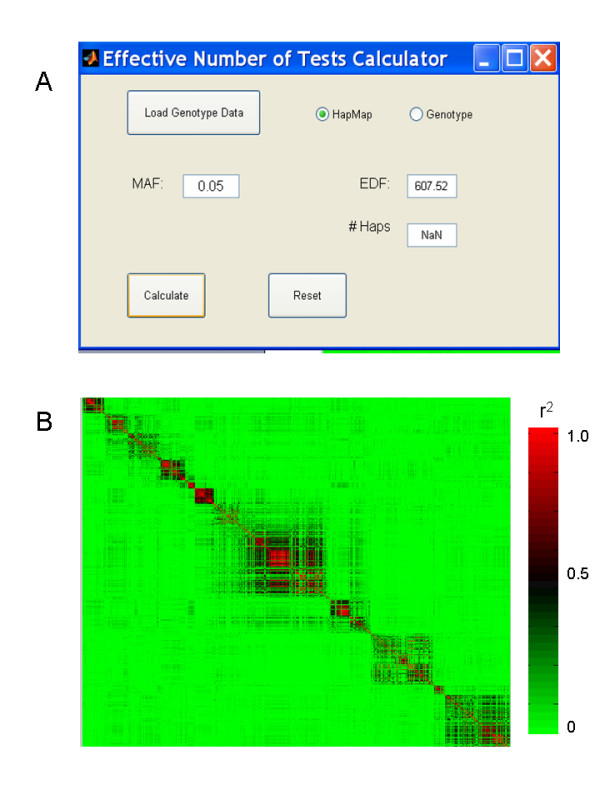
**Effective degrees of freedom calculator.** (A) HapMap SNP genotype data from human chromosome 8q24 (chr8:128100000-128700000) is used as an input. The calculated EDF for SNPs with MAF > 0.05 in this dataset is 608. (B) LD map for the selected SNPs is also displayed in the output.

An important utility for PGA1 and PGA2 is the GUI EDF, which calculates the effective degrees of freedom (EDF) for a particular set of SNP genotypes in linkage disequilibrium. This tool allows the user to assess the extent of multiple testing that is often overestimated or underestimated in naive power analyses. The EDF calculator accepts as input genotype data files from Hapmap [18] or tab-delimited text files. It calculates and maps the linkage disequilibrium patterns (r^2^) among the SNPs in the dataset, and from these data computes a summary measure of the EDF [19] (Figure 2). The value of EDF can then be used in PGA1 and PGA2 to precisely calibrate the calculations to the specific SNPs under consideration by a given study. It is important to note that other methods accounting for linkage disequilibrium between genetic markers as well as other approaches for multiple testing adjustments can be incorporated into the PGA calculations (see Additional file 1).

All the procedures included in the PGA GUIs are available in a single Matlab toolbox and can be executed at the Matlab command line. This allows Matlab users to use some of the incorporated functions in their own Matlab scripts. For example, to calculate EDF for 100 different regions with 80 SNPs each, took ~176 sec to run using a Windows XP dual 3.19 GHz, Intel Xion workstation.

## Discussion

The PGA package is well suited for power calculations where relatively small genomic regions are scanned for disease susceptibility loci. However, it can also be used to assess larger regions and even genome-wide association studies, via appropriate specification of the false positive rate, i.e. α/m where m is the number of genotyped markers in the study. Similarly to other popular software in this field [12-14], PGA incorporates basic power and sample size calculations for various genetic models and presents the results 'on the fly' in graphs and tables. In addition, it offers unique power analyses for haplotype data using the method of Chen et. al. [20]. Another novel feature is the calculation of minimal detectable risk over a range of marker allele frequencies, implemented in the PGA2 GUI. This tool may become extremely important in the current phase of genetic association studies where a large number of diseases-susceptibility genomic loci are revealed by genome-wide association studies (GWAS) [21-23]. These regions are expected to be further investigated in higher resolution, using a denser set of makers, in efforts to identify the actual predisposing genetic variation of these diseases. In this realm, PGA2 would facilitate the design of these studies by assessing power at the lower allele frequency threshold under consideration. Finally, the assessment of effective degrees of freedom for a particular genomic region or set of SNPs, as implemented in the GUI EDF, provides power calculation for procedures such as the minP test [20] that are more powerful than the conservative Bonferroni procedure. The incorporation of other methods for multiple testing adjustments (e.g. false discovery rate [24]) in automatic power calculation tools is more complex and requires specification of parameters such as the number of associated versus null SNPs and the magnitude of any effects. These calculations might be useful, especially for genome-wide association studies, but they are currently not in the scope of PGA.

Other freely-available software packages have features that are complimentary to PGA (see Additional file 2). The novel features of PGA are especially relevant to studies of candidate genes and fine-mapping efforts.

## Conclusion

The PGA package assembles a broad spectrum of statistical power calculations for genetic association studies in a single Matlab toolbox and three stand-alone GUIs. The software offers user-friendly tools for advanced calculations of statistical power and sample size and presents the results 'on the fly' in graphs and tables. Hence, PGA may significantly facilitate decision making and interpretation of association studies of candidate genes, fine-mapping studies, and genome-wide scans.

## Availability and requirements

• **Project name**: Power for genetic association analyses (PGA).

• **Project home page**: 

• **Operating system(s)**: Windows XP & Vista.

• **Programming language**: Matlab.

• **Other requirements**: To run the stand-alone GUIs, users without Matlab licenses should install first the MATLAB Component Runtime (MCR) that is available in the PGA home page.

• **Any restrictions to use by non-academics**: None

• **Reviewers access to the software**: reviewers can download the software in a way that preserves their anonymity, through the following links:

Readme file: 

PGA.exe file: .

MCRinstaller file: 

## Authors' contributions

IM drafted the manuscript and assisted in the design and implementation of the software. PSR conceived of the study, assisted in the design and implementation of the software and in drafting the manuscript. BEC developed the software and helped draft the manuscript.

## Supplementary Material

Additional file 1 Supplementary Methods. Click here for file

Additional file 2 Table 1. Major features of four commonly used power software for case-control association studies. Click here for file
