# Supplementary material for: PGA: power calculator for case-control genetic association analyses
Source: BMC Genet. 2008 May 13;9:36. doi: 10.1186/1471-2156-9-36 (PMC2387159; doi:10.1186/1471-2156-9-36)
Supplement: Additional file 1 — Supplementary Methods. [file 1471-2156-9-36-S1.pdf]

Supplemental methods

EDF calculation:

Effective degree of freedom (EDF) is calculated using the spectral decomposition of matrices of pair-wise LD between SNPs (Nyholt, 2004). The EDF value can be used for multiplicity correction in SNP-based analyses.

Power and sample-size analysis:

SNP-based analysis:

The power for SNP-based association study is computed for three genetic models (Recessive, Dominant and Co-dominant/Multiplicative) as a function of the disease-associated SNP allele frequency (p_d), marker allele frequency (p_1), the disease prevalence (ϕ), the relative risk (*R*), the type I error (α), the sample size (*n*), and the effective degrees of freedom (*EDF*). The association between the marker allele and disease allele is measured by either D' or r^2. When the input is D', given p_d and p_1, the maximum disequilibrium is $D_{\max} = \min\{p_1(1-p_d), p_d(1-p_1)\}$. Therefore, the non-scaled disequilibrium is $D = D'D_{\max}$. When the input is r^2, we have

$$r_{\max}^2 = \frac{\min\{p_1(1-p_d), p_d(1-p_1)\}^2}{p_1(1-p_d)p_d(1-p_1)}$$

and $D = \sqrt{r^2 r_{\max}^2 p_1(1-p_d)p_d(1-p_1)}$. If we let *A* denote the disease allele and *B* denote the marker allele, then the frequencies for haplotypes *AB*, *Ab*, *aB* and *ab* are

$$\begin{aligned} h_{11} &= p_d p_1 + D, \\ h_{10} &= p_d(1-p_1) - D, \\ h_{01} &= (1-p_d)p_1 - D, \\ h_{00} &= (1-p_d)(1-p_1) + D, \end{aligned}$$

respectively.

We define $F = \left[(1-p_d)^2, 2p_d(1-p_d), p_d{}^2\right]$ as the probability vector of an individual carrying 0, 1 or 2 copies of the disease causative allele and $R = [1, r_1, r_2]$ as the relative risk

vector for zero, one and two copies of the disease causative allele. Here $R=[1,1,r]$, $R=[1,r,r]$, $R=[1,r,r^2]$ are for recessive model, dominant model, co-dominant models respectively.

The disease prevalence is given by: $\phi = r_0(FR^T)$, where R^T is the transpose of the row vector R. Therefore, the baseline disease risk can be written as

$$r_0 = \frac{\phi}{FR^T}.$$

Let X_A= 0, 1, or 2 be the number of disease alleles (*A*) and X_B = 0, 1, or 2 be the number of marker alleles (*B*), and Y = 0 or 1 be the disease status. The disease penetrance for a person with $x=0$, 1, or 2 copies of marker allele is given by

$$\Pr(Y=d, X_B=x) = \sum_{j=0}^{2} \Pr(Y=d \mid X_A=j, X_B=x)\Pr(X_A=j, X_B=x)$$

$\Pr(X_A=j, X_B=x)$ is the sum of frequencies for all the haplotype pairs that have j copies of A allele and x copies of B allele:

$$\Pr(X_A=0, X_B=0) = h_{00}^2,$$
$$\Pr(X_A=0, X_B=1) = 2h_{00}h_{01}$$
$$\Pr(X_A=0, X_B=2) = h_{01}^2$$
$$\Pr(X_A=1, X_B=0) = 2h_{00}h_{10}$$
$$\Pr(X_A=1, X_B=1) = 2h_{11}h_{00} + 2h_{01}h_{10}$$
$$\Pr(X_A=1, X_B=2) = 2h_{11}h_{01}$$
$$\Pr(X_A=2, X_B=0) = h_{10}^2$$
$$\Pr(X_A=2, X_B=1) = 2h_{11}h_{10}$$
$$\Pr(X_A=2, X_B=2) = h_{11}^2$$

The exposure prevalence in cases is given by

$$P_{xd} = \Pr(X_B=x \mid Y=d) = \frac{\Pr(Y=d, X_B=x)}{\Pr(Y=d)}.$$

Therefore, the exposure prevalence in cases and controls is

$$P_{x1} = \frac{P_x}{\phi} \text{ and } P_{x0} = \frac{1-P_x}{1-\phi}, \text{ respectively.}$$

For the **dominant model**, we have the following contingency table:

Allele	$X=0$	$X=1$ or 2	Total
Cases $(D=1)$	n_1P_{01}	$n_1P_{11}+n_1P_{21}$	n_1
Controls $(D=0)$	n_0P_{00}	$n_0P_{10}+n_0P_{20}$	n_0

and the log odd ratio has asymptotic normal distribution with mean and variance

$$\mu=\log(n_0P_{00})+\log(n_1P_{11}+n_1P_{21})-\log(n_1P_{01})-\log(n_0P_{10}+n_0P_{20}),$$

$$\sigma^2=\frac{1}{n_1P_{01}}+\frac{1}{n_0P_{10}+n_0P_{20}}+\frac{1}{n_0P_{00}}+\frac{1}{n_1P_{11}+n_1P_{21}}\ .$$

All test statistics considered in the manuscript are two-sided. The power is given by:

$Pow=\Pr(|Z|>C)$, where $Z\sim N(\mu,\sigma^2)$ and $C=\Phi^{-1}\left(1-\frac{\alpha}{2EDF}\right)$.

For the **recessive model**, we have the following contingency table:

Allele	$X=0$ or 1	$X=2$	Total
Cases $(D=1)$	$n_1P_{01}+n_1P_{11}$	n_1P_{21}	n_1
Controls $(D=0)$	$n_0P_{00}+n_0P_{10}$	n_0P_{20}	n_0

and the log odd ratio has asymptotic normal distribution with mean and variance

$$\mu=\log(n_0P_{00}+n_0P_{10})+\log(n_1P_{21})-\log(n_1P_{01}+n_1P_{11})-\log(n_0P_{20}),$$

$$\sigma^2=\frac{1}{n_1P_{01}+n_0P_{10}}+\frac{1}{n_0P_{20}}+\frac{1}{n_0P_{00}+n_1P_{11}}+\frac{1}{n_1P_{21}}\ .$$

Therefore, the power is given by:

$Pow = \Pr(|Z| > C)$, where $Z \sim N(\mu, \sigma^2)$ and $C = \Phi^{-1}\left(1 - \frac{\alpha}{2EDF}\right)$.

For the **recessive model**, we have the following contingency table:

Allele	$X=0$	$X=1$	$X=2$	Total
Cases $(D=1)$	n_1P_{01}	n_1P_{11}	n_1P_{21}	n_1
Controls $(D=0)$	n_0P_{00}	n_0P_{10}	n_0P_{20}	n_0
Total	m_0	m_1	m_2	n

We use the linear trend test, which has a Chi-squared distribution with one degree of freedom under the null hypothesis. Under the alternative hypothesis, it has a non-central Chi-squared distribution with non-central parameter

$$\delta = \frac{n-1}{n} \times \frac{n_1\left\{\sum_{x=0}^{2}\left(P_{x1} - \frac{m_x}{n}\right)x\right\}^2}{n_0\left\{\sum_{x=0}^{2}\frac{m_x}{n}x^2 - \left(\sum_{x=0}^{2}\frac{m_x}{n}x\right)^2\right\}}.$$

Therefore, the power is given by $Pow = \Pr(T > C)$, where

$T \sim \chi^2_{1,\delta}$ and $C = F^{-1}_{\chi^2_1}\left(1 - \frac{\alpha}{EDF}\right)$ where $F_{\chi^2_1}(x)$ is the cumulative distribution function of a Chi-squared distribution with one degree of freedom.

Haplotype-based analysis:

For the haplotypes-based analysis we adopted the directed haplotype frequency test that compares the inferred frequencies of the common haplotypes for cases with controls (Chen, et al., 2006). The power is obtained for the three genetic models via a non-central Chi-squared distribution where the non-central parameter (8) is a function of the

frequency of disease-associated haplotypes (f), the disease prevalence (p), the relative risk (r), the type I error (α), the sample size (n) and the degree of freedom of the test (df). Let $F = [f_1, \cdots, f_K]$ be the frequency of haplotype $[h_1, \cdots h_K]$, with all the rare haplotypes being put together into the last haplotype h_K, where K = df + 1. Without lost of generality, we suppose that disease susceptibility is associated with first haplotype, which has haplotype frequency f_1. Again, the baseline disease risk can be written as

$$r_0 = \frac{\phi}{F_1 R^T},$$

where *p*, F_1 and R^T are defined as in the SNP-based analysis.

For any i and j, let $H = (h_i, h_j)$ be the haplotype pair, conditional on the disease status,

$$\begin{aligned} p_{ij}^{(1)} &= \Pr(H = (h_i, h_j) \mid Y = 1) \\ &= \frac{\Pr(Y = 1 \mid H = (h_i, h_j)) \Pr(H = (h_i, h_j))}{\Pr(Y = 1)} \\ &= \phi^{-1} r_{ij} f_i f_j, \end{aligned}$$

and

$$\begin{aligned} p_{ij}^{(0)} &= \Pr(H = (h_i, h_j) \mid D = 0) \\ &= \frac{\Pr(D = 0 \mid H = (h_i, h_j)) \Pr(H = (h_i, h_j))}{\Pr\{D = 0\}} \\ &= (1 - \phi)^{-1} (1 - r_{ij}) f_i f_j, \end{aligned}$$

where $r_{ij} = r_0 R_1$ if H contains zero copies of disease haplotype, $r_{ij} = r_0 R_2$ if H contains one copy of disease haplotype and $r_{ij} = r_0 R_3$ if H contains two copies of disease haplotype. Therefore, the haplotype frequencies in case and control groups are given by

$$f_i^{(1)} = \sum_{j=1}^{K} p_{ij}^{(1)} \text{ and } f_i^{(0)} = \sum_{j=1}^{K} p_{ij}^{(0)} .$$

The haplotype numbers in cases and controls have a multinomial distribution $\left\{n_1; f_1^{(1)}, \cdots f_K^{(1)}\right\}$ and $\left\{n_0; f_1^{(0)}, \cdots f_K^{(0)}\right\}$, respectively. By simple algebra, we have $f_i^{(1)} = af_i$ and $f_i^{(0)} = bf_i$ for $i = 2, \cdots K$, where

$$a = \phi^{-1} r_0 \left\{R_2 f_1 + R_1(1 - f_1)\right\}, \text{ and } b = (1-\phi)^{-1}\left\{(1 - r_0 R_2) f_1 + (1 - r_0 R_1)(1 - f_1)\right\}.$$

We denote $F^{(1)} = [f_2^{(1)}, \cdots, f_K^{(1)}] = a[f_2, \cdots, f_K]$ and $F^{(0)} = [f_2^{(0)}, \cdots f_K^{(0)}] = b[f_2, \cdots, f_K]$. The estimators $\widehat{F}^{(1)}$ and $\widehat{F}^{(0)}$ have mean $F^{(1)}$ and $F^{(0)}$, respectively. The corresponding variance-covariance matrix is given by

$$S_1 = n_1^{-1}\left\{diag\left(F^{(1)}\right) - \left(F^{(1)}\right)^T \times F^{(1)}\right\} \text{ and } S_0 = n_0^{-1}\left\{diag\left(F^{(0)}\right) - \left(F^{(0)}\right)^T \times F^{(0)}\right\}.$$

Under the null hypothesis, the directed haplotype frequency test statistic T has a Chi-squared distribution with $K-1$ degrees of freedom. Under the alternative hypothesis, it has a non-central Chi-squared distribution with non-central parameter $\delta = dV^{-1}d^T$, where $d = (a-b)\mathbf{f}$, $\mathbf{f} = [f_2, \cdots, f_K]$ and

$$V = S_1 + S_0 = \left(\frac{a}{n_1} + \frac{b}{n_0}\right) diag(\mathbf{f}) - \left(\frac{a^2}{n_1} + \frac{b^2}{n_0}\right)\mathbf{f}^T\mathbf{f}.$$

Then we have

$$V^{-1} = \left(\frac{a}{n_1} + \frac{b}{n_0}\right)^{-1}\left\{diag(\mathbf{f})^{-1} + \frac{c}{1 - c(1 - f_1)}\mathbf{1}^T\mathbf{1}\right\}$$

where $c = \dfrac{n_0 a^2 + n_1 b^2}{n_0 a + n_1 b}$, $\mathbf{1} = [1, \cdots, 1]$.

Finally the power of the directed haplotype frequency test is given by:

$Pow = \Pr(T > C)$, where $T \sim \chi^2_{K-1,\delta}$ and $C = \chi^2_{K-1}\left(1-\alpha\right)$.

We now show that that the non-central parameter and the power depend only on the frequency of the disease haplotype f_1,

$$
\begin{aligned}
\delta &= dV^{-1}d^T \\
&= tr(dV^{-1}d^T) \\
&= \frac{n_1 n_0 (a-b)^2}{n_0 a + n_1 b} tr\left\{ \mathbf{f} diag(\mathbf{f})^{-1} \mathbf{f}' - \frac{c}{1-c(1-f_1)} \mathbf{f} \mathbf{1}^T \mathbf{1} \mathbf{f}^T \right\} \\
&= \frac{n_1 n_0 (a-b)^2}{n_0 a + n_1 b} \left\{ tr(diag(\mathbf{f})) - \frac{c}{1-c(1-f_1)} tr(\mathbf{f}\mathbf{1}^T)^2 \right\} \\
&= \frac{n_1 n_0 (a-b)^2}{n_0 a + n_1 b} \left\{ (1-f_1) - \frac{c(1-f_1)^2}{1-c(1-f_1)} \right\}.
\end{aligned}
$$

Since here a, b and c only depend on haplotype frequency f_1, the power depends only on f_1.

Minimal detectable relative risk calculation:

For the calculation of the minimal detectable relative risk we used the *fzero* function of Matlab® that tries to find the zero of the power functions described above within a given relative risk interval. For the model with different relative risks associate with different copies of disease allele or haplotype, we fixed the ratio of the two relative risks and find the minimal detectable relative risk for containing one copy of disease allele or haplotype.

References:

Chen, B.E., Sakoda, L.C., Hsing, A.W. and Rosenberg, P.S. (2006) Resampling-based multiple hypothesis testing procedures for genetic case-control association studies, *Genetic epidemiology*, **30**, 495-507.

Nyholt, D.R. (2004) A simple correction for multiple testing for single-nucleotide polymorphisms in linkage disequilibrium with each other, *American journal of human genetics*, **74**, 765-769.
