# Supplementary material for: PGA: power calculator for case-control genetic association analyses
Source: BMC Genet. 2008 May 13;9:36. doi: 10.1186/1471-2156-9-36 (PMC2387159; doi:10.1186/1471-2156-9-36)
Supplement: Additional file 2 — Table 1. Major features of four commonly used power software for case-control association studies. [file 1471-2156-9-36-S2.pdf]

Table 1. Major features of four commonly used power software for case-control association studies.

Software name	GPC	CaTS	PAWE 3D	PGA
URL	http://pngu.mgh.harvard.edu/~purcell/gpc/cc2.html	http://www.sph.umich.edu/csg/abecasis/CaTS/	http://linkage.rockefeller.edu/pawe3d/pawe3dmain.html	http://dceg.cancer.gov/bb/tools/pga
Web-based	√		√	
Downloadable GUI		√		√
Power and sample size calculations for SNP data	√	√	√	√
Power and sample size calculations for haplotype data				√
Power and sample size calculations for multi-stages studies		√		
Accounts for linkage disequilibrium between marker and causative allele			√	√
Calculate minimal detectable relative risk (MDRR)				√
Analyze power for a range of parameters			√	√
Has a graphical output		√	√	√
Power and sample size calculations for gene-gene interactions	√			
Accounts for phenotypic and genotypic errors			√	
Available as editable code				√
